# Multiple Maternal Chronic Conditions and Risk of Severe Neonatal Morbidity and Mortality

**DOI:** 10.1001/jamanetworkopen.2025.55558

**Published:** 2026-01-23

**Authors:** Hilary K. Brown, Kinwah Fung, Eyal Cohen, Cindy-Lee Dennis, Sonia M. Grandi, Laura C. Rosella, Catherine Varner, Simone N. Vigod, Jannah Wigle, Walter P. Wodchis, Joel G. Ray

**Affiliations:** 1 Department of Health and Society, University of Toronto Scarborough, Toronto, Ontario, Canada; 2 Dalla Lana School of Public Health, University of Toronto, Toronto, Ontario, Canada; 3 Women’s College Research Institute, Women’s College Hospital, Toronto, Ontario, Canada; 4 ICES, Toronto, Ontario, Canada; 5 Institute of Health Policy, Management and Evaluation, University of Toronto, Toronto, Ontario, Canada; 6 Hospital for Sick Children, Toronto, Ontario, Canada; 7 Edwin S.H. Leong Centre for Healthy Children, Toronto, Ontario, Canada; 8 Department of Pediatrics, Temerty Faculty of Medicine, University of Toronto, Toronto, Ontario, Canada; 9 Lawrence Bloomberg Faculty of Nursing, University of Toronto, Toronto, Ontario, Canada; 10 Lunenfeld-Tanenbaum Research Institute, Mount Sinai Hospital, Toronto, Ontario, Canada; 11 Institute for Better Health, Trillium Health Partners, Mississauga, Ontario, Canada; 12 Department of Laboratory Medicine and Pathobiology, Temerty Faculty of Medicine, University of Toronto, Toronto, Ontario, Canada; 13 Mount Sinai Hospital, Toronto, Ontario, Canada; 14 Department of Family and Community Medicine, Temerty Faculty of Medicine, University of Toronto, Toronto, Ontario, Canada; 15 Department of Psychiatry, Temerty Faculty of Medicine, University of Toronto, Toronto, Ontario, Canada; 16 Li Ka Shing Knowledge Institute, Unity Health Toronto, Toronto, Ontario, Canada

## Abstract

**Importance:**

Nearly 16% of pregnant individuals have multiple chronic conditions (MCC), and the risk of severe maternal morbidity and mortality increases in a dose-response manner with the number of preexisting conditions. However, little is known about newborn outcomes in this population.

**Objective:**

To examine the association of the number of preexisting maternal chronic conditions, as well as the presence of MCC complexity, cardiometabolic MCC, and MCC severity, with the risk of severe neonatal morbidity or mortality (SNM-M).

**Design, Setting, and Participants:**

This population-based cohort study was conducted in Ontario, Canada, among all live births from 2012 to 2021. Data were analyzed from September 2024 to November 2025.

**Exposure:**

Maternal MCC measured in the 2 years before conception.

**Main Outcomes and Measures:**

Modified Poisson regression was performed to generate adjusted relative risks (aRRs) for SNM-M by the number of chronic conditions, MCC complexity (≥3 chronic conditions in ≥3 body systems), co-occurring cardiometabolic conditions, and MCC severity marked by a prenatal hospitalization for a chronic illness. Multivariable models were adjusted for age, parity, immigration status, income quintile, and rurality.

**Results:**

The cohort comprised 1 018 968 newborns, including 20 934 to mothers with 3 or more chronic conditions (mean [SD] maternal age, 30.0 [6.3] years), 73 768 to mothers with 2 chronic conditions (mean [SD] maternal age, 30.3 [5.8] years), 276 765 to mothers with 1 chronic condition (mean [SD] maternal age, 30.7 [5.4] years), and 647 501 to mothers with 0 chronic conditions (mean [SD] maternal age, 31.0 [5.1] years). Compared with newborns of mothers with 0 chronic conditions, the aRR for SNM-M increased in a dose-response fashion in newborns of mothers with 1 (1.26; 95% CI, 1.24-1.28), 2 (1.58; 95% CI, 1.54-1.62), and 3 or more (2.01; 95% CI, 1.94-2.09) chronic conditions. The aRRs were also increased with complex MCC (1.97; 95% CI, 1.88-2.06), cardiometabolic MCC (2.67; 95% CI, 2.24-3.19), and severe MCC (3.11; 95% CI, 2.55-3.79).

**Conclusions and relevance:**

In this study, risks of SNM-M increased with an increasing number of preexisting maternal chronic conditions. These findings suggest that women and adolescents with MCC may benefit from preconception counseling to optimize chronic disease management, monitoring in pregnancy for earlier identification of complications, and enhanced newborn supports.

## Key Points

**Question:**

What is the association of the number, complexity, type, and severity of preexisting maternal chronic conditions with severe neonatal morbidity or mortality?

**Findings:**

In this cohort study of 1 018 968 births, there was a dose-response association between the number of maternal chronic conditions before pregnancy and the risk of severe neonatal morbidity or mortality, preterm birth, and congenital anomalies.

**Meaning:**

These findings suggest the importance of preconception counseling for mothers with multiple chronic conditions to optimize chronic disease management, monitoring in pregnancy for earlier identification of complications, and enhanced newborn supports.

## Teaser

This cohort study investigates the association of the number, complexity, severity, and cardiometabolic status of multiple maternal chronic conditions (MCC) with severe neonatal morbidity or mortality.

## Introduction

The prevalence of chronic conditions, such as diabetes, depression, and cardiovascular disease, is increasing, impacting nearly half the global population.^1^ Adults with multiple chronic conditions (MCC) have disproportionately high health care costs, with elevated acute care use and premature mortality.^2,3^ While patients with MCC have typically been studied within geriatric and primary care settings,^4,5^ emerging evidence suggests that consideration of MCC is important in maternal-newborn care. Nearly 16% of pregnant women have MCC,^6^ and risks of maternal morbidity and mortality increase in a dose-response manner with the number of chronic conditions.^7,8,9,10^ That risk is thought to be partly due to challenges in managing several chronic conditions within health care systems that are designed to address single and acute conditions, along with the abnormal physiology brought on by MCC and increased likelihood of drug-drug interactions.^7,8,9,10^

While maternal health has been considered in association with MCC, few studies have explored the association with infant outcomes, such as severe neonatal morbidity and mortality (SNM-M).^11^ Three studies found a dose-response association between the number of prepregnancy chronic conditions and risk of preterm birth.^7,12,13^ One examined severe outcomes, showing increased odds of neonatal readmission and mortality in newborns of women with 2 or more vs 0 chronic conditions, although the latter finding was nonsignificant.^12^ Understanding contributors to SNM-M is important to identify how to prevent such outcomes, which have a substantial burden on newborns, families, and the health system. Little is known about the risk of SNM-M associated with maternal MCC and how risks may vary with MCC complexity, type, and severity. For example, we showed that MCC were associated with a greater increase in severe maternal morbidity and mortality when MCC comprised disparate, rather than closely related conditions, possibly due to complexity of disease management across several specialties.^10^ Cardiometabolic MCC due to co-occurring diabetes, chronic hypertension, cardiac disease, or obesity, which each pose pregnancy risk, were also associated with more adverse maternal outcomes than other MCC.^9^ Whether such associations persist for neonatal outcomes is unknown. Also unexplored is the role of MCC severity.

Having better data on the risk of SNM-M in women with MCC can inform preconception counseling, along with better planning of prenatal visits, labor and birth, and newborn care. Accordingly, this study examined the risk of SNM-M by the number of prepregnancy chronic conditions, as well as by MCC complexity, cardiometabolic type, and severity.

## Methods

### Study Design and Data Sources

This population-based cohort study was completed in Ontario, Canada, using administrative data from ICES (formerly, the Institute for Clinical Evaluative Sciences) and the Better Outcomes Registry and Network. Ontario has a universal health care plan wherein residents receive essential medical services at no direct cost. We accessed and analyzed datasets (eTable 1 in Supplement 1) at ICES, linked using unique encoded identifiers.^14^ ICES is a prescribed entity under Ontario’s Personal Health Information Protection Act (PHIPA). Section 45 of PHIPA authorizes ICES to collect personal health information without consent for health care planning. Data use was authorized under section 45 and approved by the University of Toronto ethics board. This study is reported following the Strengthening the Reporting of Observational Studies in Epidemiology (STROBE) reporting guideline.

### Cohort

The cohort comprised Ontarian women and adolescent girls aged 13 to 54 years with a singleton live birth at 20 weeks’ or later gestation between April 1, 2012, and March 31, 2021. Excluded were births to women and adolescents who did not have provincial health insurance in the 2-year period before conception, such as someone who was not a resident of Ontario for the 2 years (eFigure 1 in Supplement 1).

In the primary analyses, maternal MCC was defined based on the number of recorded chronic condition diagnoses 2 years before conception.^10^ We began with a list of diagnoses used in previous MCC research, including 16 chronic conditions selected based on their frequency and cost to the health care system.^15,16,17^ We then added another 6 conditions specifically relevant to pregnancy.^18^ The final list of 22 conditions was as follows: alcohol and substance use disorders, asthma, cancer, cardiac arrhythmia, chronic hypertension, chronic liver disease, chronic obstructive pulmonary disease, congestive heart failure, coronary artery syndrome, diabetes, HIV, inflammatory bowel disease, kidney failure, migraine, mood and anxiety disorders, multiple sclerosis, obesity, osteoarthritis, other mental illness, rheumatoid arthritis, stroke, and systemic lupus erythematosus. These conditions were ascertained via validated algorithms where available (eTable 2 in Supplement 1).^19,20,21,22,23,24,25,26,27,28,29,30,31,32,33,34,35,36,37,38^ From this list, we counted the number of conditions mothers had during their 2-year lookback window and categorized them as having 0, 1, 2, or 3 or more conditions.

In secondary analyses, we examined MCC complexity, type, and severity. Complex MCC was defined as having 3 or more chronic conditions affecting 3 or more body systems,^39,40^ and mothers were categorized as having 0 chronic conditions, 1 chronic condition, complex MCC, or noncomplex MCC.^10^ Next, cardiometabolic MCC comprised cardiac arrhythmia, chronic hypertension, congestive heart failure, coronary artery syndrome, diabetes, obesity, and stroke,^41^ with a mother categorized as having 0 cardiometabolic or noncardiometabolic chronic conditions; 1, 2, or 3 or more cardiometabolic conditions; or 1, 2, or 3 or more noncardiometabolic conditions.^10^ Finally, a nonbirth hospitalization for chronic disease during pregnancy may be a proxy for severity.^42^ Thus, for the third secondary analysis, we categorized a mother as having 0 chronic conditions; 1, 2, or 3 or more conditions with a concomitant hospitalization for the chronic condition during the pregnancy; or 1, 2, or 3 or more conditions without a prenatal hospitalization for a chronic condition.

### Outcomes

The primary outcome was SNM-M. SNM is a near-miss composite associated with neonatal mortality, length of stay, and readmission, measured using the Neonatal Adverse Outcomes Indicator.^43^ This validated measure includes 14 diagnoses (eg, seizure) and 7 procedure codes (eg, newborn resuscitation) recorded during the birth admission and less than 28 days after discharge.^43^ Mortality was also measured during the birth admission up to less than 28 days after discharge. Secondary outcomes comprised other neonatal adversity indicators that often occur in parallel with SNM-M: spontaneous and clinician-initiated preterm birth at less than 37 weeks gestation^44^ and major congenital anomalies, the latter diagnosed up to age 1 year.^45^

### Covariates

Following the perinatal health framework of Misra et al,^46^ covariates were sociodemographic factors that differed between mothers with and without MCC and that may be associated with neonatal risk, namely maternal age, parity, immigration status (Immigration, Refugees, and Citizenship Canada Permanent Residents Database), neighborhood income quintile, and rural residence.^47^ We did not adjust for assisted reproductive technologies, maternal complications, or lifestyle behaviors, which could be on the causal pathway between MCC and SNM-M (eFigure 2 in Supplement 1).

### Statistical Analysis

Baseline characteristics of mothers with 1, 2, and 3 or more chronic conditions vs 0 chronic conditions were described and compared using standardized differences.^48^ For primary analyses, modified Poisson regression^49^ was used to generate relative risks (RRs) and 95% CIs for SNM-M and secondary outcomes, comparing newborns of mothers with 1, 2, and 3 or more antecedent chronic conditions with those with 0 conditions (the reference group). Generalized estimating equations^50^ accounted for potentially more than 1 newborn per mother during the study period. RRs were adjusted for covariates listed previously. We also examined the risk of SNM-M and other adverse neonatal outcomes associated with maternal MCC complexity, the clustering of cardiometabolic chronic conditions, and being hospitalized during pregnancy for a chronic condition as a marker of MCC severity.

In additional analyses, we expanded the chronic condition lookback period to up to 10 years to ensure that we captured mothers with conditions who may not have sought health care for the condition proximal to pregnancy. Second, we added several rare conditions (ie, cardiomyopathy, congenital heart disease, and sickle cell disease) to our definition of MCC; these conditions are not frequently used in measures of MCC but may be relevant to pregnancy.^51^ Third, the number of medications a mother is prescribed may be another measure of MCC.^51^ Hence, SNM-M and secondary outcomes were reevaluated in newborns of mothers prescribed 1, 2, and 3 or more vs 0 medications 2 years before conception. Each medication was counted once, and repeat prescriptions or those for different doses or formulations of the same medicines were not recounted.^52,53^ This analysis was limited to a subset of pregnant mothers who were eligible for free medication coverage under the Ontario Drug Benefit Program, which is provided to individuals who have high prescription drug costs, are unemployed, receive home care services, or have a disability.^54^ Finally, we used multinomial logistic regression to study the odds of 1 and 2 or more vs 0 SNM-M indicators in mothers with 1, 2, and 3 or more chronic conditions compared with mothers with 0 conditions. Analyses were performed using SAS statistical software version 9.4 (SAS Institute Inc). Statistical significance was determined by 95% CIs that did not cross 1. Data were analyzed from September 2024 to November 2025.

## Results

### Main Findings

During the study period, there were 1 018 968 births, including 20 934 (2.1%) to mothers with 3 or more chronic conditions (mean [SD] maternal age [SD], 30.0 [6.3] years) 73 768 (7.2%) to mothers with 2 chronic conditions (mean [SD] maternal age, 30.3 [5.8] years), 276 765 (27.2%) to mothers with 1 chronic condition (mean [SD] maternal age, 30.7 [5.4] years), and 647 501 (63.5%) to mothers with 0 chronic conditions (mean [SD] maternal age, 31.0 [5.1] years) (Table 1). In contrast to mothers with 0 chronic conditions, mothers with MCC were younger, less likely to be immigrants, and more likely to reside within a low-income neighborhood and a rural area.

**Table 1.** Study Population Characteristics

Baseline characteristic	Mothers with live births, No. (%) (N = 1 018 968)						
	0 Conditions (n = 647 501)	1 Condition (n = 276 765)	Standardized difference, 1 vs 0 conditions	2 Conditions (n = 73 768)	Standardized difference, 2 vs 0 conditions	≥3 Conditions (n = 20 934)	Standardized difference, ≥3 vs 0 conditions
Age, y							
Mean (SD)	31.0 (5.1)	30.7 (5.4)	0.07	30.3 (5.8)	0.13	30.0 (6.3)	0.19
13-24	67 396 (10.4)	37 748 (13.6)	0.10	12 747 (17.3)	0.20	4444 (21.2)	0.30
25-34	420 704 (65.0)	171 607 (62.0)	0.06	42 740 (57.9)	0.14	11 251 (53.7)	0.23
35-44	157 741 (24.4)	66 620 (24.1)	0.01	18 027 (24.4)	0.00	5146 (24.6)	0.01
45-54	1660 (0.3)	790 (0.3)	0.01	254 (0.3)	0.02	93 (0.4)	0.03
Multiparous	366 193 (56.6)	162 091 (58.6)	0.04	43 322 (58.7)	0.04	12 259 (58.6)	0.04
Immigrant status							
Long-term resident	494 586 (76.4)	235 021 (84.9)	0.22	65 269 (88.5)	0.32	18 897 (90.3)	0.38
Immigrant	133 536 (20.6)	34 289 (12.4)	0.22	6765 (9.2)	0.33	1557 (7.4)	0.39
Refugee	19 379 (3.0)	7455 (2.7)	0.02	1734 (2.4)	0.04	480 (2.3)	0.04
Neighborhood income quintile							
First (lowest)	123 207 (19.0)	62 314 (22.5)	0.09	19 636 (26.6)	0.18	6686 (31.9)	0.30
Second	124 781 (19.3)	56 391 (20.4)	0.03	15 991 (21.7)	0.06	4585 (21.9)	0.07
Third	136 949 (21.2)	58 017 (21.0)	0.00	14 599 (19.8)	0.03	3915 (18.7)	0.06
Fourth	142 960 (22.1)	56 165 (20.3)	0.04	13 627 (18.5)	0.09	3389 (16.2)	0.15
Fifth (highest)	117 996 (18.2)	43 045 (15.6)	0.07	9717 (13.2)	0.14	2286 (10.9)	0.21
Missing	1608 (0.2)	833 (0.3)	0.01	198 (0.3)	0.00	73 (0.3)	0.02
Rural residence							
Yes	60 872 (9.4)	33 338 (12.0)	0.09	9268 (12.6)	0.10	2704 (12.9)	0.11
Missing	791 (0.1)	349 (0.1)	0.00	88 (0.1)	0.00	36 (0.2)	0.01

Compared with newborns of mothers with 0 chronic conditions (40 205 newborns with SNM-M [6.2%]), the risk of SNM-M increased in a dose-response fashion among newborns of mothers with 1 (21 722 newborns [7.8%]; adjusted RR [aRR], 1.26; 95% CI, 1.24-1.28), 2 (7347 newborns [10.0%]; aRR, 1.58; 95% CI, 1.54-1.62), and 3 or more (2672 newborns [12.8%]; aRR, 2.01; 95% CI, 1.94-2.09) chronic conditions. Similar patterns were seen for spontaneous and clinician-initiated preterm birth and congenital anomalies (Table 2).

**Table 2.** Risk of Adverse Neonatal Outcomes by No. of Prepregnancy Chronic Conditions

Outcome by No. maternal chronic conditions	Newborns		RR (95% CI)	
	Total, No.	With outcome, No. (%)	Unadjusted	Adjusted^a^
**SNM-M <28 d**				
0 Chronic conditions	647 501	40 205 (6.2)	1 [Reference]	1 [Reference]
1 Chronic condition	276 765	21 722 (7.8)	1.26 (1.24-1.28)	1.26 (1.24-1.28)
2 Chronic conditions	73 768	7347 (10.0)	1.59 (1.55-1.63)	1.58 (1.54-1.62)
≥3 Chronic conditions	20 934	2672 (12.8)	2.03 (1.96-2.11)	2.01 (1.94-2.09)
**Spontaneous preterm birth <37 wk gestation**				
0 Chronic conditions	647 501	20 073 (3.1)	1 [Reference]	1 [Reference]
1 Chronic condition	276 765	9900 (3.6)	1.14 (1.12-1.17)	1.13 (1.11-1.16)
2 Chronic conditions	73 768	3327 (4.5)	1.42 (1.37-1.48)	1.40 (1.35-1.45)
≥3 Chronic conditions	20 934	1300 (6.2)	1.92 (1.82-2.04)	1.86 (1.76-1.97)
**Clinician-initiated preterm birth <37 wk gestation**				
0 Chronic conditions	647 501	20 019 (3.1)	1 [Reference]	1 [Reference]
1 Chronic condition	276 765	11 680 (4.2)	1.35 (1.32-1.38)	1.36 (1.33-1.39)
2 Chronic conditions	73 768	4273 (5.8)	1.83 (1.77-1.89)	1.84 (1.78-1.90)
≥3 Chronic conditions	20 934	1806 (8.6)	2.69 (2.57-2.82)	2.70 (2.58-2.84)
**Any congenital anomaly**				
0 Chronic conditions	647 501	32 927 (5.1)	1 [Reference]	1 [Reference]
1 Chronic condition	276 765	16 032 (5.8)	1.14 (1.12-1.16)	1.12 (1.10-1.15)
2 Chronic conditions	73 768	4910 (6.7)	1.30 (1.27-1.34)	1.29 (1.25-1.32)
≥3 Chronic conditions	20 934	1539 (7.4)	1.44 (1.37-1.51)	1.42 (1.35-1.49)

Abbreviations: RR, relative risk; SNM-M, severe neonatal morbidity or mortality.

^a^ Adjusted for maternal age, parity, immigrant and refugee status, neighborhood income quintile, and rural residence.

Compared with newborns of mothers with 0 chronic conditions, the aRR for SNM-M was more pronounced for newborns of mothers with complex MCC (1.97; 95% CI, 1.88-2.06) than those with noncomplex MCC (1.62; 95% CI, 1.59-1.66). The same was seen for aRRs of spontaneous and clinician-initiated preterm birth and congenital anomalies (Table 3).

**Table 3.** Risk of Adverse Neonatal Outcomes by Prepregnancy MCC Complexity

Outcome by maternal MCC complexity	Newborns		RR (95% CI)	
	Total, No.	With outcome, No. (%)	Unadjusted	Adjusted^a^
**SNM-M <28 d**				
0 Chronic conditions	647 501	40 205 (6.2)	1 [Reference]	1 [Reference]
1 Chronic condition	276 765	21 722 (7.8)	1.26 (1.24-1.28)	1.26 (1.24-1.28)
Noncomplex MCC	80 738	8285 (10.3)	1.64 (1.60-1.67)	1.62 (1.59-1.66)
Complex MCC	13 964^b^	1734 (12.4)	1.98 (1.89-2.07)	1.97 (1.88-2.06)
**Spontaneous preterm birth <37 wk**				
0 Chronic conditions	647 501	20 073 (3.1)	1 [Reference]	1 [Reference]
1 Chronic condition	276 765	9900 (3.6)	1.14 (1.12-1.17)	1.13 (1.11-1.16)
Noncomplex MCC	80 738	3830 (4.7)	1.49 (1.44-1.54)	1.46 (1.41-1.51)
Complex MCC	13 964^b^	797 (5.7)	1.77 (1.65-1.91)	1.73 (1.61-1.86)
**Clinician-initiated preterm birth <37 wk**				
0 Chronic conditions	647 501	20 019 (3.1)	1 [Reference]	1 [Reference]
1 Chronic condition	276 765	11 680 (4.2)	1.35 (1.32-1.38)	1.36 (1.33-1.39)
Noncomplex MCC	80 738	4888 (6.1)	1.91 (1.85-1.97)	1.92 (1.86-1.98)
Complex MCC	13 964^b^	1191 (8.5)	2.66 (2.51-2.81)	2.65 (2.50-2.81)
**Any congenital anomaly**				
0 Chronic conditions	647 501	32 927 (5.1)	1 [Reference]	1 [Reference]
1 Chronic condition	276 765	16 032 (5.8)	1.14 (1.12-1.16)	1.12 (1.10-1.15)
Noncomplex MCC	80 738	5425 (6.7)	1.32 (1.28-1.35)	1.30 (1.26-1.33)
Complex MCC	13 964^b^	1024 (7.3)	1.44 (1.35-1.53)	1.42 (1.33-1.51)

Abbreviations: MCC, multiple chronic conditions; RR, relative risk; SNM-M, severe neonatal morbidity or mortality.

^a^ Adjusted for maternal age, parity, immigrant and refugee status, neighborhood income quintile, and rural residence.

^b^ Complex MCC was defined as the co-occurrence of 3 or more chronic conditions affecting 3 or more body systems, with systems defined by chapters of the *International Statistical Classification of Diseases and Related Health Problems, Tenth Revision* (*ICD-10*).

There was an increasing risk of SNM-M by the number of maternal cardiometabolic and noncardiometabolic conditions, with the largest aRR seen in the presence of several cardiometabolic conditions. Compared with newborns of mothers with 0 chronic conditions, the aRR for SNM-M was 2.67 (95% CI, 2.24-3.19) for newborns of mothers with 3 or more cardiometabolic conditions and 1.94 (95% CI, 1.81-2.08) for newborns of mothers with 3 or more noncardiometabolic conditions (Table 4). Similar patterns were seen for spontaneous and clinician-initiated preterm birth, with the greatest magnitude of risk for clinician-initiated preterm birth, and congenital anomalies (Table 4).

**Table 4.** Risk of Adverse Neonatal Outcomes by No. of Cardiometabolic and Noncardiometabolic Chronic Conditions

Outcome by No. maternal cardiometabolic or noncardiometabolic chronic conditions	Newborns		RR (95% CI)	
	Total, No.	With outcome, No. (%)	Unadjusted	Adjusted^a^
**SNM-M <28 d**				
0 Chronic conditions	647 501	40 205 (6.2)	1 [Reference]	1 [Reference]
1 Noncardiometabolic condition	145 221	11 216 (7.7)	1.24 (1.21-1.26)	1.23 (1.20-1.25)
2 Noncardiometabolic conditions	27 381	2640 (9.6)	1.54 (1.48-1.60)	1.52 (1.46-1.58)
≥3 Noncardiometabolic conditions	6018	743 (12.3)	1.96 (1.83-2.10)	1.94 (1.81-2.08)
1 Cardiometabolic condition	183 819	15 802 (8.6)	1.38 (1.35-1.40)	1.38 (1.35-1.40)
2 Cardiometabolic conditions	8453	1237 (14.6)	2.32 (2.20-2.45)	2.26 (2.14-2.38)
≥3 Cardiometabolic conditions	575	103 (17.9)	2.82 (2.36-3.36)	2.67 (2.24-3.19)
**Spontaneous preterm birth <37 wk**				
0 Chronic conditions	647 501	20 073 (3.1)	1 [Reference]	1 [Reference]
1 Noncardiometabolic condition	145 221	5861 (4.0)	1.28 (1.24-1.31)	1.26 (1.23-1.30)
2 Noncardiometabolic conditions	27 381	1533 (5.6)	1.73 (1.64-1.83)	1.69 (1.60-1.78)
≥3 Noncardiometabolic conditions	6018	426 (7.1)	2.14 (1.94-2.36)	2.05 (1.85-2.26)
1 Cardiometabolic condition	183 819	6177 (3.4)	1.08 (1.05-1.11)	1.07 (1.04-1.10)
2 Cardiometabolic conditions	8453	486 (5.7)	1.78 (1.63-1.95)	1.76 (1.61-1.93)
≥3 Cardiometabolic conditions	575	44 (7.7)	2.39 (1.79-3.19)	2.34 (1.75-3.12)
**Clinician-initiated preterm birth <37 wk**				
0 Chronic conditions	647 501	20 019 (3.1)	1 [Reference]	1 [Reference]
1 Noncardiometabolic condition	145 221	5884 (4.1)	1.29 (1.26-1.33)	1.31 (1.27-1.35)
2 Noncardiometabolic conditions	27 381	1450 (5.3)	1.66 (1.57-1.75)	1.71 (1.62-1.81)
≥3 Noncardiometabolic conditions	6018	436 (7.2)	2.26 (2.05-2.48)	2.38 (2.17-2.62)
1 Cardiometabolic condition	183 819	8860 (4.8)	1.54 (1.50-1.58)	1.53 (1.50-1.57)
2 Cardiometabolic conditions	8453	1017 (12.0)	3.70 (3.48-3.94)	3.37 (3.16-3.58)
≥3 Cardiometabolic conditions	575	112 (19.5)	5.95 (5.00-7.08)	5.07 (4.26-6.04)
**Any congenital anomaly**				
0 Chronic conditions	647 501	32 927 (5.1)	1 [Reference]	1 [Reference]
1 Noncardiometabolic condition	145 221	8190 (5.6)	1.11 (1.08-1.13)	1.09 (1.07-1.12)
2 Noncardiometabolic conditions	27 381	1734 (6.3)	1.24 (1.18-1.30)	1.22 (1.17-1.28)
≥3 Noncardiometabolic conditions	6018	428 (7.1)	1.39 (1.27-1.53)	1.37 (1.25-1.50)
1 Cardiometabolic condition	183 819	11 359 (6.2)	1.21 (1.19-1.24)	1.20 (1.17-1.22)
2 Cardiometabolic conditions	8453	716 (8.5)	1.65 (1.54-1.78)	1.62 (1.51-1.74)
≥3 Cardiometabolic conditions	575	54 (9.4)	1.83 (1.41-2.37)	1.78 (1.37-2.31)

Abbreviations: RR, relative risk; SNM-M, severe neonatal morbidity or mortality.

^a^ Adjusted for maternal age, parity, immigrant and refugee status, neighborhood income quintile, and rural residence.

In the assessment of MCC severity, the most pronounced risk was seen for newborns of mothers who had MCC and a concomitant prenatal hospitalization for a chronic condition. Compared with mothers with 0 chronic conditions and no hospitalization in pregnancy, the aRR for SNM-M was 3.11 (95% CI, 2.55-3.79) for mothers with 3 or more conditions and a prenatal hospitalization and 1.99 (95% CI, 1.92-2.07) for mothers with 3 or more conditions but no prenatal hospitalization (Table 5).

**Table 5.** Risk of Adverse Neonatal Outcomes by Hospitalization During Pregnancy for Chronic Condition

Outcome by No. maternal chronic conditions and whether hospitalized during pregnancy for those conditions^a^	Newborns		RR (95% CI)	
	Total, No.	With outcome, No. (%)	Unadjusted	Adjusted^b^
**SNM-M <28 d**				
0 Chronic conditions and no hospitalization in pregnancy	647 501	40 205 (6.2)	1 [Reference]	1 [Reference]
1 Chronic condition and no hospitalization in pregnancy	276 407	21 672 (7.8)	1.26 (1.24-1.28)	1.25 (1.23-1.27)
2 Chronic conditions and no hospitalization in pregnancy	73 398	7297 (9.9)	1.59 (1.55-1.62)	1.58 (1.54-1.62)
≥3 Chronic conditions and no hospitalization in pregnancy	20 528	2592 (12.6)	2.01 (1.93-2.09)	1.99 (1.92-2.07)
1 Chronic condition and ≥1 hospitalizations in pregnancy	358	50 (14.0)	2.22 (1.71-2.87)	2.19 (1.69-2.83)
2 Chronic conditions and ≥1 hospitalizations in pregnancy	370	50 (13.5)	2.17 (1.68-2.81)	2.13 (1.65-2.76)
≥3 Chronic conditions and ≥1 hospitalizations in pregnancy	406	80 (19.7)	3.13 (2.57-3.82)	3.11 (2.55-3.79)
**Spontaneous preterm birth <37 wk**				
0 Chronic conditions and no hospitalization in pregnancy	647 501	20 073 (3.1)	1 [Reference]	1 [Reference]
1 Chronic condition and no hospitalization in pregnancy	276 407	9872 (3.6)	1.14 (1.11-1.17)	1.13 (1.11-1.16)
2 Chronic conditions and no hospitalization in pregnancy	73 398	3300 (4.5)	1.42 (1.37-1.47)	1.39 (1.34-1.45)
≥3 Chronic conditions and no hospitalization in pregnancy	20 528	1252 (6.1)	1.89 (1.78-2.00)	1.83 (1.73-1.94)
1 Chronic condition and ≥1 hospitalizations in pregnancy	358	28 (7.8)	2.39 (1.66-3.46)	2.28 (1.58-3.29)
2 Chronic conditions and ≥1 hospitalizations in pregnancy	370	27 (7.3)	2.35 (1.64-3.35)	2.21 (1.55-3.15)
≥3 Chronic conditions and ≥1 hospitalizations in pregnancy	406	48 (11.8)	3.66 (2.78-4.82)	3.45 (2.62-4.55)
**Clinician-initiated preterm birth <37 wk**				
0 Chronic conditions and no hospitalization in pregnancy	647 501	20 019 (3.1)	1 [Reference]	1 [Reference]
1 Chronic condition and no hospitalization in pregnancy	276 407	11 648 (4.2)	1.35 (1.32-1.38)	1.36 (1.33-1.39)
2 Chronic conditions and no hospitalization in pregnancy	73 398	4239 (5.8)	1.82 (1.76-1.88)	1.83 (1.77-1.89)
≥3 Chronic conditions and no hospitalization in pregnancy	20 528	1756 (8.6)	2.67 (2.54-2.80)	2.68 (2.55-2.81)
1 Chronic condition and ≥1 hospitalizations in pregnancy	358	32 (8.9)	2.72 (1.94-3.82)	2.85 (2.03-4.01)
2 Chronic conditions and ≥1 hospitalizations in pregnancy	370	34 (9.2)	2.95 (2.15-4.04)	3.06 (2.23-4.19)
≥3 Chronic conditions and ≥1 hospitalizations in pregnancy	406	50 (12.3)	3.82 (2.93-4.97)	4.06 (3.11-5.30)
**Any congenital anomaly**				
0 Chronic conditions and no hospitalization in pregnancy	647 501	32 927 (5.1)	1 [Reference]	1 [Reference]
1 Chronic condition and no hospitalization in pregnancy	276 407	16 009 (5.8)	1.14 (1.12-1.16)	1.12 (1.10-1.15)
2 Chronic conditions and no hospitalization in pregnancy	73 398	4889 (6.7)	1.31 (1.27-1.34)	1.29 (1.25-1.33)
≥3 Chronic conditions and no hospitalization in pregnancy	20 528	1508 (7.3)	1.44 (1.37-1.51)	1.42 (1.35-1.49)
1 Chronic condition and ≥1 hospitalizations in pregnancy	358	23 (6.4)	1.25 (0.84-1.86)	1.23 (0.83-1.84)
2 Chronic conditions and ≥1 hospitalizations in pregnancy	370	21 (5.7)	1.11 (0.73-1.69)	1.10 (0.72-1.67)
≥3 Chronic conditions and ≥1 hospitalizations in pregnancy	406	31 (7.6)	1.50 (1.07-2.10)	1.48 (1.06-2.08)

Abbreviations: RR, relative risk; SNM-M, severe neonatal morbidity or mortality.

^a^ Mothers are categorized by number of chronic conditions and whether they were hospitalized during pregnancy for that condition or those conditions.

^b^ Adjusted for maternal age, parity, immigrant and refugee status, neighborhood income quintile, and rural residence.

### Additional Results

The aRRs were similar to those in main models when we extended the lookback period to up to 10 years. However, the overall prevalence of MCC increased (eTable 3 in Supplement 1). Findings were also similar when we added cardiomyopathy, congenital heart disease, and sickle cell disease to the definition of MCC (eTable 4 in Supplement 1).

The risk of SNM-M was greater in newborns of mothers who used 2 or 3 or more medications in pregnancy vs no medication, but the risk was not greater in newborns of those using just 1 medication. The same pattern was observed for spontaneous and clinician-initiated preterm birth. There was no risk of congenital anomalies until at least 3 medications were prescribed (eTable 5 in Supplement 1). Finally, newborns of mothers with 1, 2, and 3 or more chronic conditions had progressively increased odds of 1 and 2 or more SNM-M indicators (eTable 6 in Supplement 1).

## Discussion

### Summary

In this cohort study, a dose-response association was observed between the number of preexisting maternal chronic conditions and risks of SNM-M and other adverse neonatal outcomes. The greatest increases in risks were among newborns whose mothers had complex or cardiometabolic MCC and those with severe MCC. These findings suggest that mothers with MCC may benefit from preconception counseling, monitoring in pregnancy for earlier identification of complications, and enhanced newborn supports.

### Comparison to Prior Research

A handful of studies have explored neonatal outcomes in women with MCC. Using the US National Inpatient Sample, Admon et al^7^ defined MCC based on the presence of 2 or more conditions among diabetes, heart disease, HIV, hypertension, kidney disease, respiratory disease, and substance use disorders. They found an increasing risk of preterm birth associated with 0 (5.7%), 1 (9.7%), and 2 or more (15.1%) maternal chronic conditions.^7^ In an analysis of the Japan Environment and Children’s Study, Nakanishi et al^13^ identified MCC based on the presence of 2 or more conditions among allergic diseases, anemia, diabetes, dyslipidemia, epilepsy, gastric or duodenal ulcer, heart disease, hepatitis, HIV, hypertension, inflammatory bowel disease, kidney disease, malignant neoplasm, migraine, neurological disease, sexually transmitted infections, psychiatric disorders, rheumatic or collagen diseases, and thyroid disease. They found an increased risk of preterm birth (adjusted odds ratio [aOR], 1.50; 95% CI, 1.33-1.69) and small for gestational age (aOR, 1.33; 95% CI 1.20-1.46) in newborns of women with 2 or more chronic conditions vs none. Likewise, using Scottish Morbidity Records, Azcoaga-Lorenzo et al^12^ defined MCC using 79 physical and mental health conditions and observed increased odds of preterm birth (aOR, 1.64; 95% CI, 1.48-1.82) in the presence of 2 or more vs 0 maternal chronic conditions. They also found increased odds of neonatal readmission (aOR, 1.35; 95% CI, 1.19-1.53) and mortality (aOR, 1.28; 95% CI, 0.64–2.59), with the latter model being underpowered.^12^ Despite heterogeneous measures of MCC, studies have been similar in their findings and support the results of our study. Our findings provide additional information about the association between MCC and SNM-M, including the more specific associations of MCC complexity, cardiometabolic MCC, and severe MCC. Findings on these patterns complement our prior study of MCC and risk of severe maternal morbidity and mortality, which showed similar patterns.^10^

### Potential Explanations for Findings

Each chronic condition included in the definition of MCC is associated with risk for fetal or neonatal adversity.^18^ There are plausible reasons why the co-occurrence of such conditions would potentiate the risk of SNM-M and other complications. Outside of pregnancy, patients with MCC experience dysregulated physiology and an increased tendency for drug-drug interactions.^2,3,4,5^ In maternal MCC, such factors are expected to influence placental health, fetal growth, a predisposition to preterm birth, and thus greater neonatal adversity. Our additional analyses and secondary outcomes can inform hypotheses about plausible pathways of association. For example, risks of SNM-M and other adverse outcomes were greatest in newborns of mothers with complex and severe MCC, suggesting that more complicated or uncontrolled MCC may have downstream associations with placental and fetal health. Moreover, the increase in the risk of preterm birth, especially clinician-initiated preterm birth, with an increasing number of chronic conditions, is evidence that the problem precedes the birth. Future studies should examine the interplay between MCC and placentation, fetal growth, and superimposed maternal conditions, such as preeclampsia.

Studies also suggest greater psychosocial and health care demands in managing multiple chronic conditions.^2,3,4,5^ In pregnancy specifically, qualitative studies indicate significant challenges for women with MCC navigating obstetric and other specialist health services.^55^ Future studies should examine women with MCC and their experiences with care continuity and coordination, health care professional communication, and participation in decision-making throughout the perinatal period, including decisions related to birth and newborn care.

### Limitations

This study has several limitations. The study included nearly all births in Ontario. However, we required individuals to have 2 years of Ontario health insurance before delivery to accurately measure chronic conditions, which excluded women who migrated to Ontario during this period. Results may not be generalizable to recent refugees and immigrants. Likewise, prescription medication data were available only for individuals eligible for the Ontario Drug Benefit Program; results may not be generalizable to the entire obstetric population.

We ascertained most chronic conditions using validated algorithms,^19,20,21,22,23,24,25,26,27,28,29,30,31,32,33,34,35,36,37,38^ but algorithms captured only patients who sought care for their condition during the defined lookback period. We may have misclassified individuals facing health care barriers or whose conditions were well-managed and not the focus of care. The measure of MCC as an indicator of multimorbidity was not validated, and not all chronic conditions were included. However, we chose conditions based on those used frequently in MCC studies.^15,16,17^ Selected conditions are common, are associated with high health care costs,^15,16,17^ and contribute the most to multimorbidity.^16^ Moreover, findings were similar when we added rare conditions relevant to pregnancy (ie, cardiomyopathy, congenital heart disease, and sickle cell disease)^51^ to the definition of MCC. We were unable to measure the severity of conditions by self-reported, clinical, or laboratory measures. However, obstetric comorbidity indices that weight risk factors by severity include both preexisting conditions and pregnancy-related complications.^51^ Our study focuses on prepregnancy MCC, so we used chronic condition–related hospital admissions as a proxy for severity.

Data on congenital anomalies are largely missing for stillbirths in Ontario administrative data, so we restricted this analysis (and others) to live births. While this restriction may have resulted in underestimation of the population prevalence of congenital anomalies, stillbirths are rare and their exclusion likely did not have a major impact on results.

We lacked data on individual-level socioeconomic status and race and ethnicity as a proxy for experiences of racism. Failure to account for these confounders may have resulted in overestimation of observed risks. We did not adjust for assisted reproductive technology, smoking, substance use, or maternal complications given that these may be on the causal pathway between MCC and SNM-M. Also unmeasured were care by maternal-fetal medicine, cardiology, or other subspecialties before or during pregnancy, as well as prenatal care quality. Future studies should examine potential mediators of the association between MCC and SNM-M.

## Conclusions

This cohort study offers novel information for clinicians, including pregnancy care clinicians, pediatricians, and family physicians. The association of MCC with the risk of neonatal complications reinforces the importance of reducing risk factors for chronic illness, including poverty, physical inactivity, and poor nutrition, and optimizing prepregnancy chronic disease management in the preconception period. Given that 40% of pregnancies are unintended,^56^ preconception care is critical for reducing risks before conception, rather than at the first prenatal care visit, when it may be too late to address exposures relevant to adverse neonatal outcomes, such as congenital anomalies.^57^ Prenatal care for women with MCC requires a multidisciplinary approach,^58^ including obstetric, medical, anesthesiology, and pediatric specialists,^58^ and a proactive care plan for labor, birth, and newborn care. Given the risks of prematurity and neonatal morbidity in women with MCC, this plan should include delivery at a hospital equipped with higher-level neonatal care and prearrangement of postdischarge home care supports.^57^ Finally, long-term developmental outcomes should be studied in children born to women with MCC.

## Notes

Supplement 1. **eFigure 1.** Study flowchart **eFigure 2.** Conceptual framework **eTable 1.** ICES datasets included in the study **eTable 2.** Approach for defining each study exposure condition and associated chronic condition prevalence. **eTable 3.** Risk of severe neonatal morbidity or mortality (SNM-M) and other adverse neonatal outcomes in mothers with 0, 1, 2, or ≥3 prepregnancy chronic conditions using a lookback period of up to 10 y **eTable 4.** Risk of severe neonatal morbidity or mortality (SNM-M) and other adverse neonatal outcomes in mothers with 0, 1, 2, or ≥3 prepregnancy chronic conditions, additionally including cardiomyopathy, congenital heart disease, and sickle cell disease in the list of chronic conditions **eTable 5.** Risk of severe neonatal morbidity or mortality (SNM-M) and other adverse neonatal outcomes in relation to the number of prescribed medications in the 2-y period before the index pregnancy conception date **eTable 6.** Odds of 1 and ≥2 indicators of severe neonatal morbidity or mortality (SNM-M) in mothers with 0, 1, 2, or ≥3 prepregnancy chronic conditions

Supplement 2.
Data Sharing Statement
