# Supplementary material for: Multiple Maternal Chronic Conditions and Risk of Severe Neonatal Morbidity and Mortality
Source: JAMA Netw Open. 2026 Jan 23;9(1):e2555558. doi: 10.1001/jamanetworkopen.2025.55558 (PMC12831157; doi:10.1001/jamanetworkopen.2025.55558)
Supplement: Supplement 1. — eFigure 1. Study flowchart eFigure 2. Conceptual framework eTable 1. ICES datasets included in the study eTable 2. Approach for defining each study exposure condition and associated chronic condition prevalence. eTable 3. Risk of severe neonatal morbidity or mortality (SNM-M) and other adverse neonatal outcomes in mothers with 0, 1, 2, or ≥3 prepregnancy chronic conditions using a lookback period of up to 10 y eTable 4. Risk of severe neonatal morbidity or mortality (SNM-M) and other adverse neonatal outcomes in mothers with 0, 1, 2, or ≥3 prepregnancy chronic conditions, additionally including cardiomyopathy, congenital heart disease, and sickle cell disease in the list of chronic conditions eTable 5. Risk of severe neonatal morbidity or mortality (SNM-M) and other adverse neonatal outcomes in relation to the number of prescribed medications in the 2-y period before the index pregnancy conception date eTable 6. Odds of 1 and ≥2 indicators of severe neonatal morbidity or mortality (SNM-M) in mothers with 0, 1, 2, or ≥3 prepregnancy chronic conditions [file jamanetwopen-e2555558-s001.pdf]

# Supplemental Online Content

Brown HK, Fung K, Cohen E, et al. Multiple maternal chronic conditions and risk of severe neonatal morbidity and mortality. *JAMA Netw Open*. 2026;9(1):e2555558. doi:10.1001/jamanetworkopen.2025.55558

**eFigure 1.** Study flowchart

**eFigure 2.** Conceptual framework

**eTable 1.** ICES datasets included in the study

**eTable 2.** Approach for defining each study exposure condition and associated chronic condition prevalence.

**eTable 3.** Risk of severe neonatal morbidity or mortality (SNM-M) and other adverse neonatal outcomes in mothers with 0, 1, 2, or ≥3 prepregnancy chronic conditions using a lookback period of up to 10 y

**eTable 4.** Risk of severe neonatal morbidity or mortality (SNM-M) and other adverse neonatal outcomes in mothers with 0, 1, 2, or ≥3 prepregnancy chronic conditions, additionally including cardiomyopathy, congenital heart disease, and sickle cell disease in the list of chronic conditions

**eTable 5.** Risk of severe neonatal morbidity or mortality (SNM-M) and other adverse neonatal outcomes in relation to the number of prescribed medications in the 2-y period before the index pregnancy conception date

**eTable 6.** Odds of 1 and ≥2 indicators of severe neonatal morbidity or mortality (SNM-M) in mothers with 0, 1, 2, or ≥3 prepregnancy chronic conditions

This supplemental material has been provided by the authors to give readers additional information about their work.

© 2026 Brown HK et al. *JAMA Network Open*.

**eFigure 1. Study flowchart**.

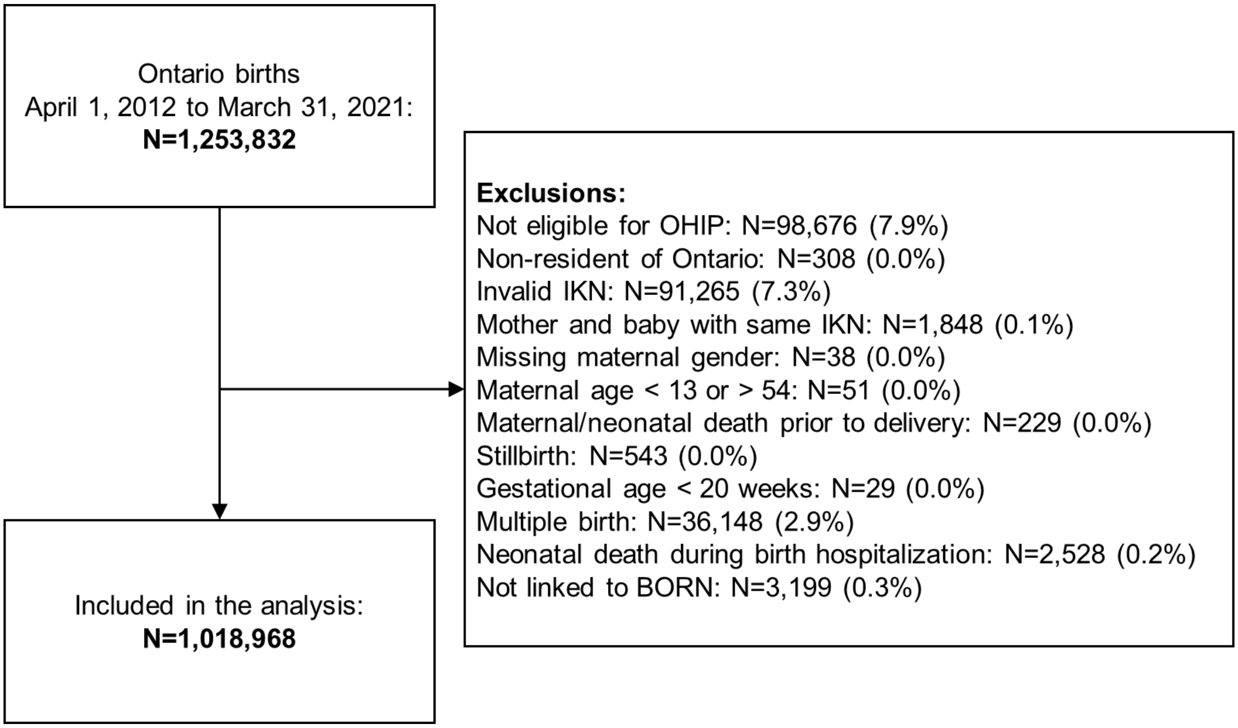


© 2026 Brown HK et al. *JAMA Network Open*.

**eFigure 2. Conceptual Framework.** Grey = not measurable in health administrative data.

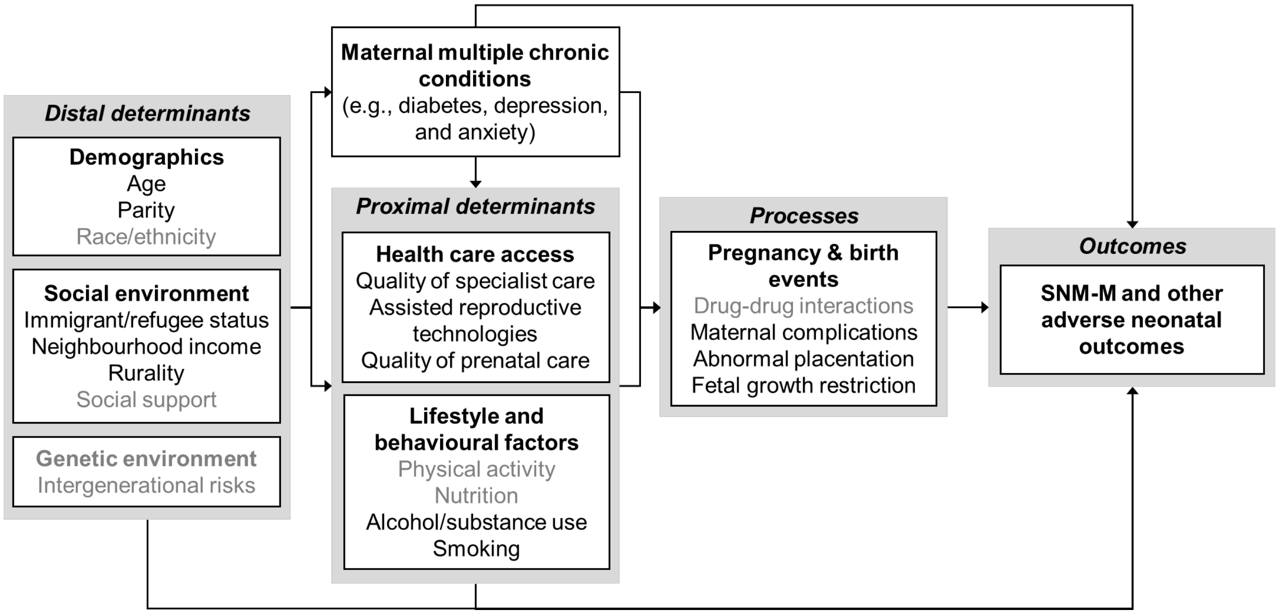


© 2026 Brown HK et al. *JAMA Network Open*.

## eTable 1. ICES datasets included in the study.

| Data source | Variable | Coding structure |
|---|---|---|
| Canadian Institute for Health Information Discharge Abstract Database | Hospital admission | Canadian Coding Standards for the International Classification of Diseases and Related Health Problems codes for diagnoses and Canadian Classification of Health Interventions codes for procedures |
| Census | Sociodemographic data | N/A |
| Immigrants, Refugees, and Citizenship Canada Permanent Residents Database | Immigration status | N/A |
| National Ambulatory Care Reporting System | Emergency department visits | Canadian Coding Standards for the International Classification of Diseases and Related Health Problems codes for diagnoses and Canadian Classification of Health Interventions codes for procedures |
| Ontario Drug Benefits Database | Prescription | Drug identification number |
| Ontario Health Insurance Database | Outpatient physician visits | Physician billing codes |
| Ontario Mental Health Reporting System | Psychiatric hospital admissions | Diagnostic and Statistical Manual of Mental Disorders |
| Registered Persons Database | Sociodemographic data | N/A |

© 2026 Brown HK et al. *JAMA Network Open*.

**eTable 2. Approach for defining each study exposure condition, and associated chronic condition prevalence.**

| Condition | Algorithm | Body system | Overall prevalence, No. (%) |
|---|---|---|---|
| Alcohol and substance use disorders | 2 physician visits: Psychiatrist [SPEC=19] and outpatient (LOCATION: O, L, H, P) and non-lab service [substr(FEECODE,1,1) ne 'G'] OR Family physician / general practitioner / pediatrician [SPEC=00 or SPEC=26] and mental illness or addiction diagnosis code [DXCODE] and outpatient (LOCATION: O, L, H, P) and non-lab service [substr(FEECODE,1,1) ne 'G'] OR pediatrician [SPEC=26] and undefined location (LOCATION = U) and MHA diagnostic code [DXCODE] and fee code (FEECODE=K122 or K123 or K704), where DXCODES = 291, 292, 303, 304; OR 1 hospital admission or ED visit: CIHI-DAD/NACRS: DX10CODE1 F10-F19, F55; OMHRS: Before 2016/17: AXIS1_DSM4CODE_DISCH1 = 291.x (all 291 codes, excluding 291.82), 292.x (all 292 codes, excluding 292.85), 303.x (all 303 codes), 304.x (all 304 codes), 305.x (all 305 codes), PROVDX_DSM4CODE_ADM1: 4; 2016/17-2018/19: DSM5CODE_DISCH1 = 291.x (all 291 codes), 292.x (all 292 codes), 303.x (all 303 codes), 304.x (all 304 codes), 305.x, Provisional = 16; 2019/20 to present: ICD10CMCODE_DISCH1=F10-F19, Z72.0; Provisional = 16[20] | Mental | 15,301 (1.5) |
| Asthma | 2 physician visits (OHIP: 493) or 1 hospital admission (ICD-10: J45)[21] | Respiratory | 34,558 (3.4) |
| Cancer | 2 physician visits (OHIP: 140-239) or 1 hospital admission or ED visit (ICD-10: C00-C26, C30-C97)[22] | Neoplasms | 7,785 (0.8) |
| Cardiac arrhythmia | 4 physician visits (OHIP: 427) (separated by 30 days) or 1 hospital admission or ED visit (ICD-10: I48.0, I48.1)[23] | Circulatory | 3,688 (0.4) |
| Chronic hypertension | 1 hospital admission (ICD: I10-I13, I15) or 1 physician visit (OHIP: 401-405) followed by an additional physician visit or hospital admission (excluding gestational hypertension)[24] | Circulatory | 9,843 (1.0) |
| Chronic liver disease | 1 physician visit (OHIP: 571) or 1 hospital admission or ED visit (ICD-10: K70.0, K70.2, K70.3, K71.7, K73, K74.6, K75.4, K75.8, K75.9, K76.0, B18)[25] | Digestive | 1,420 (0.1) |
| Chronic obstructive pulmonary disease | 1 physician visit (OHIP: 491, 492, 496) or 1 hospital admission (ICD-10: J41, J43, J44) and 35 years or older[26] | Respiratory | 843 (0.1) |
| Congestive heart failure | 1 physician visit (OHIP: 428 or feecode Q050) or 1 hospital admission (ICD-10: I50.0, I50.1, I50.9) followed by a 2nd[27] | Circulatory | 19 (0.0) |
| Coronary artery syndrome | 2 physician visits (OHIP: 411-414) or 1 hospital admission or ED visit (I20-I25)[28] | Circulatory | 1,348 (0.1) |
| Diabetes | If < 19 years: 4 physician visits (OHIP: 250) or procedures (Q040, K029, K030, K045, K046) within 2 years and at least 1 of these before 19th birthday; if ≥ 19 years: 2 | Endocrine and metabolic | 9,781 (1.0) |

© 2026 Brown HK et al. *JAMA Network Open*.

| Condition | Algorithm | Body system | Overall prevalence, No. (%) |
|---|---|---|---|
| | physician visits (OHIP: 250) or 1 procedure (Q040, K029, K030, K045, K046) or hospital admission (ICD-10: E10, E11, E13, E14) (excluding gestational diabetes)[29] | | |
| HIV | 3 physician visits (OHIP: 042-044) or 1 hospital admission (ICD-10: B20-B24)[30] | Infectious | 473 (0.0) |
| Inflammatory bowel disease | 2 years of OHIP eligibility and 5 physician visits (OHIP: 555, 556) or hospital admission or ED visits (ICD-10: K50, K51) OR < 2 years of OHIP eligibility and 3 physician visits (OHIP: 555, 556) or hospital admission or ED visits (ICD-10: K50, K51)[31] | Digestive | 5,307 (0.5) |
| Migraine | 2 physician visits (OHIP: 346) or 1 hospital admission or ED visit (ICD-9ICD-10: G43, G44, G97.1, N95.1, R51)[32] | Nervous | 36,753 (3.6) |
| Mood or anxiety disorders | 2 physician visits with a Psychiatrist [SPEC=19] and outpatient (LOCATION: O, L, H, P) and non-lab service [substr(FEECODE,1,1) ne 'G'] OR Family physician / general practitioner / pediatrician [SPEC=00 or SPEC=26] and mental illness or addiction diagnosis code [DXCODE] and outpatient (LOCATION: O, L, H, P) and non-lab service [substr(FEECODE,1,1) ne 'G'] OR pediatrician [SPEC=26] and undefined location (LOCATION = U) and MHA diagnostic code [DXCODE] and fee code (FEECODE=K122 or K123 or K704), where DXCODES = 296, 300, 309, 311; OR 1 hospital admission or ED visit: CIHI-DAD/NACRS: Before 2016/17: DX10CODE1 F30-F34, F38-F43, F48.8, F48.9, F53.0, F93.1-F93.2; 2016/17-present: DX10CODE1 F06.3, F06.4, F30-F34, F38-F43, F45.2, F53.0, F63.3, F93.0-F93.2, F94.0-F94.2; OMHRS: Before 2016/17: AXIS1_DSM4CODE_DISCH1 = 296.x (all 296 codes), 300, 300.0x, 300.2x, 300.3x, 300.4x, 301.13, 308.3x, 309.0x, 309.24, 309.28, 309.3x, 309.4x, 309.8x, 309.9x, 311; Provisional: 6, 7, 15. 2016/17-2018/19: DSM5CODE_DISCH1 = 293.83, 293.84, 296.x (all 296 codes), 300, 300.0x, 300.2x, 300.3x, 300.4x, 300.7x, 301.13, 308.3x, 309, 309.0x, 309.21, 309.24, 309.28, 309.3x, 309.4x, 309.81, 309.89, 309.9x, 311.x, 312.39, 313.23, 313.89, 625.4, 698.4x, Provisional = 3-7; 2019/20 to present: ICD10CMCODE_DISCH1=F06.3, F06.4, F06.8, F31-F34, F40.0-F40.2, F41.0, F41.1, F41.8, F41.9, F42.2-F42.4, F42.8, F42.9, F43.0-F43.2, F43.8, F43.9, F45.2, F63.3, F91.4, F94.2, F93.0, F94.0-F94.2; Provisional = 3-7[20] | Mental | 135,177 (13.3) |
| Multiple sclerosis | 5 physician visits (OHIP: 340) or 1 hospital admission or ED visit (ICD-10: G35)[33] | Nervous | 1,147 (0.1) |
| Obesity | If linked to BORN: BMI – NIDAY period = BMI: Overweight/obese ≥ 30.0, Other < 30.0; BMI – BIS period: MATERNAL_BMI = Overweight/obese ≥ 30.0; Other < 25.0. Where BMI/MATERNAL_BMI is missing: 2 physician visits (OHIP: 278) or 1 ED visit or hospital admission (ICD-10: E66)[10] | Endocrine and metabolic | 177,276 (17.4) |
| Osteoarthritis | 2 physician visits (OHIP: 715) or 1 hospital admission or ED visit (ICD-10: M00-M03, M07, M10, M11-M25, M30-36, M65-M79)[34] | Musculoskeletal and connective tissue | 26,963 (2.6) |

© 2026 Brown HK et al. *JAMA Network Open*.

| Condition | Algorithm | Body system | Overall prevalence, No. (%) |
|---|---|---|---|
| Other mental illness | 2 physician visits: Psychiatrist [SPEC=19] and outpatient (LOCATION: O, L, H, P) and non-lab service [substr(FEECODE,1,1) ne 'G'] OR Family physician / general practitioner / pediatrician [SPEC=00 or SPEC=26] and mental illness or addiction diagnosis code [DXCODE] and outpatient (LOCATION: O, L, H, P) and non-lab service [substr(FEECODE,1,1) ne 'G'] OR pediatrician [SPEC=26] and undefined location (LOCATION = U) and MHA diagnostic code [DXCODE] and fee code (FEECODE=K122 or K123 or K704), where DXCODES = 301, 302, 306, 307, 309, 313-315; OR 1 hospital admission or ED visit: CIHI-DAD/NACRS: DX10CODE1 All other F06-F99 from MHAP algorithm not included in other categories, and excluding IDD diagnoses (F70-F73, F78, F79, F84.0, F84.1, F84.3-F84.9); OMHRS: All other OMHRS from MHAP algorithm not included in other categories, and excluding IDD diagnoses[20] | Mental | 16,864 (1.7) |
| Psychotic mental illness | 2 physician visits: Psychiatrist [SPEC=19] and outpatient (LOCATION: O, L, H, P) and non-lab service [substr(FEECODE,1,1) ne 'G'] OR Family physician / general practitioner / pediatrician [SPEC=00 or SPEC=26] and mental illness or addiction diagnosis code [DXCODE] and outpatient (LOCATION: O, L, H, P) and non-lab service [substr(FEECODE,1,1) ne 'G'] OR pediatrician [SPEC=26] and undefined location (LOCATION = U) and MHA diagnostic code [DXCODE] and fee code (FEECODE=K122 or K123 or K704), where DXCODES = 295, 297, 298; OR 1 hospital admission or ED visit: CIHI-DAD/NACRS: DX10CODE1 F20 (excluding F20.4), F22-F25, F28-F29, F53.1; OMHRS: Before 2016/17: AXIS1_DSM4CODE_DISCH1 = 295.x (all 295 codes), 297.x (all 297 codes), 298.x (all 298 codes); Provisional = 5; 2016/17-2018/19: DSM5CODE_DISCH1 = 293.81, 293.82, 295.x (all 295 codes), 297.x (all 297 codes), 298.x (all 298 codes), Provisional = 2; 2019/20 to present: ICD10CMCODE_DISCH1=F20.81, F20.9, F22, F23, F25, F06.0-F06.2, F28, F29; Provisional = 2.[35] | Mental | 1,918 (0.2) |
| Renal failure | 1 physician visit (OHIP: 403, 404, 584, 585, 586) or hospital admission (ICD-10: N17, N18, N19, T82.4, Z49.2, Z99.2)[36] | Genitourinary | 2,051 (0.2) |
| Rheumatoid arthritis | 3 physician visits (OHIP: 714) with ≥ 1 by a specialist (rheumatologist, internal medicine specialist, orthopedic surgeon) or 1 hospital admission or ED visit (ICD-10: M05, M06)[37] | Musculoskeletal and connective tissue | 2,156 (0.2) |
| Stroke | 2 physician visits (OHIP: 430-434, 436) or 1 hospital admission or ED visit (ICD-10: I60-I64)[38] | Circulatory | 523 (0.1) |
| Systemic lupus erythematosus | 3 physician visits (OHIP: 710) with ≥ 1 by a specialist (rheumatologist) or 1 hospital admission (ICD-10: M32)[39] | Musculoskeletal and connective tissue | 1,463 (0.1) |

© 2026 Brown HK et al. *JAMA Network Open*.

Abbreviations: CIHI-DAD = Canadian Institute for Health Information Discharge Abstract Database; DSM = Diagnostic and Statistical Manual of Mental Disorders; ICD = International Classification of Diseases and Related Health Problems; NACRS = National Ambulatory Care Reporting System; OHIP = Ontario Health Insurance Plan; OMHRS = Ontario Mental Health Reporting System

© 2026 Brown HK et al. *JAMA Network Open*.

**eTable 3. Risk of severe neonatal morbidity or mortality (SNM-M) and other adverse neonatal outcomes, in mothers with 0, 1, 2 or ≥ 3 pre-pregnancy chronic conditions, using a lookback period of up to 10 years.**

| **Study outcome *by number of chronic conditions*** | **No. (%) with outcome** | **Unadjusted relative risk (95% CI)** | **Adjusted relative risk (95% CI)[a]** |
|---|---|---|---|
| **Severe neonatal morbidity or mortality < 28 days** | | | |
| *0 chronic conditions (N=408,052)* | 24,239 (5.9) | 1.00 (ref.) | 1.00 (ref.) |
| *1 chronic condition (N=317,821)* | 21,815 (6.9) | 1.15 (1.13-1.17) | 1.16 (1.14-1.18) |
| *2 chronic conditions (N=172,109)* | 13,829 (8.0) | 1.35 (1.32-1.38) | 1.35 (1.33-1.38) |
| *≥ 3 chronic conditions (N=120,986)* | 12,063 (10.0) | 1.67 (1.64-1.71) | 1.69 (1.65-1.72) |
| | | | |
| **Spontaneous preterm birth < 37 weeks' gestation** | | | |
| *0 chronic conditions (N=408,052)* | 11,963 (2.9) | 1.00 (ref.) | 1.00 (ref.) |
| *1 chronic condition (N=317,821)* | 10,220 (3.2) | 1.09 (1.06-1.12) | 1.10 (1.07-1.13) |
| *2 chronic conditions (N=172,109)* | 6,355 (3.7) | 1.25 (1.21-1.29) | 1.25 (1.21-1.29) |
| *≥ 3 chronic conditions (N=120,986)* | 6,062 (5.0) | 1.68 (1.63-1.73) | 1.66 (1.61-1.72) |
| | | | |
| **Provider-initiated preterm birth < 37 weeks' gestation** | | | |
| *0 chronic conditions (N=408,052)* | 11,783 (2.9) | 1.00 (ref.) | 1.00 (ref.) |
| *1 chronic condition (N=317,821)* | 11,013 (3.5) | 1.20 (1.17-1.23) | 1.21 (1.18-1.25) |
| *2 chronic conditions (N=172,109)* | 7,410 (4.3) | 1.48 (1.44-1.52) | 1.52 (1.48-1.57) |
| *≥ 3 chronic conditions (N=120,986)* | 7,572 (6.3) | 2.14 (2.08-2.20) | 2.22 (2.15-2.28) |
| | | | |
| **Any congenital anomaly** | | | |
| *0 chronic conditions (N=408,052)* | 20,107 (4.9) | 1.00 (ref.) | 1.00 (ref.) |
| *1 chronic condition (N=317,821)* | 17,124 (5.4) | 1.09 (1.07-1.11) | 1.08 (1.05-1.10) |
| *2 chronic conditions (N=172,109)* | 10,154 (5.9) | 1.20 (1.17-1.23) | 1.17 (1.15-1.20) |
| *≥ 3 chronic conditions (N=120,986)* | 8,023 (6.6) | 1.34 (1.31-1.38) | 1.32 (1.29-1.35) |

Abbreviations: CI = confidence interval.

[a] Adjusted for maternal age, parity, immigrant/refugee status, neighbourhood income quintile, and rural residence.

© 2026 Brown HK et al. *JAMA Network Open*.

**eTable 4. Risk of severe neonatal morbidity or mortality (SNM-M) and other adverse neonatal outcomes, in mothers with 0, 1, 2 or ≥ 3 pre-pregnancy chronic conditions, additionally including cardiomyopathy, congenital heart disease, and sickle cell disease in the list of chronic conditions.**

| Study outcome *by number of chronic conditions* | No. (%) with outcome | Unadjusted relative risk (95% CI) | Adjusted relative risk (95% CI)[a] |
|---|---|---|---|
| **Severe neonatal morbidity or mortality < 28 days** | | | |
| *0 chronic conditions (N=646,618)* | 40,151 (6.2) | 1.00 (ref.) | 1.00 (ref.) |
| *1 chronic condition (N=277,221)* | 21,733 (7.8) | 1.26 (1.24-1.28) | 1.25 (1.23-1.27) |
| *2 chronic conditions (N=74,065)* | 7,379 (10.0) | 1.59 (1.55-1.63) | 1.58 (1.54-1.62) |
| *≥ 3 chronic conditions (N=21,064)* | 2,683 (12.7) | 2.03 (1.95-2.10) | 2.01 (1.93-2.08) |
| | | | |
| **Spontaneous preterm birth < 37 weeks' gestation** | | | |
| *0 chronic conditions (N=646,618)* | 20,037 (3.1) | 1.00 (ref.) | 1.00 (ref.) |
| *1 chronic condition (N=277,221)* | 9,919 (3.6) | 1.14 (1.12-1.17) | 1.13 (1.11-1.16) |
| *2 chronic conditions (N=74,065)* | 3,336 (4.5) | 1.42 (1.37-1.48) | 1.40 (1.35-1.45) |
| *≥ 3 chronic conditions (N=21,064)* | 1,308 (6.2) | 1.92 (1.82-2.04) | 1.86 (1.76-1.97) |
| | | | |
| **Provider-initiated preterm birth < 37 weeks' gestation** | | | |
| *0 chronic conditions (N=646,618)* | 19,980 (3.1) | 1.00 (ref.) | 1.00 (ref.) |
| *1 chronic condition (N=277,221)* | 11,697 (4.2) | 1.35 (1.32-1.38) | 1.36 (1.33-1.39) |
| *2 chronic conditions (N=74,065)* | 4,283 (5.8) | 1.82 (1.77-1.88) | 1.84 (1.78-1.90) |
| *≥ 3 chronic conditions (N=21,064)* | 1,818 (8.6) | 2.69 (2.57-2.82) | 2.71 (2.58-2.84) |
| | | | |
| **Any congenital anomaly** | | | |
| *0 chronic conditions (N=646,618)* | 32,867 (5.1) | 1.00 (ref.) | 1.00 (ref.) |
| *1 chronic condition (N=277,221)* | 16,066 (5.8) | 1.14 (1.12-1.16) | 1.13 (1.10-1.15) |
| *2 chronic conditions (N=74,065)* | 4,927 (6.7) | 1.30 (1.27-1.34) | 1.29 (1.25-1.33) |
| *≥ 3 chronic conditions (N=21,064)* | 1,548 (7.3) | 1.44 (1.37-1.51) | 1.42 (1.35-1.49) |

Abbreviations: CI = confidence interval.

[a] Adjusted for maternal age, parity, immigrant/refugee status, neighbourhood income quintile, and rural residence.

© 2026 Brown HK et al. *JAMA Network Open*.

**eTable 5. Risk of severe neonatal morbidity or mortality (SNM-M) and other adverse neonatal outcomes, in relation to the number of prescribed medications in the two-year period before the index pregnancy conception date.** This analysis is limited to a sub-cohort of 142,442 pregnancies among mothers eligible for free medication cost coverage under the Ontario Drug Benefit Program.

| **Study outcome *by number of prescribed medications*** | **No. (%) with outcome** | **Unadjusted relative risk (95% CI)** | **Adjusted relative risk (95% CI)[a]** |
|---|---|---|---|
| **Severe neonatal morbidity or mortality < 28 days** | | | |
| *0 medications (N=48,482)* | 3,927 (8.1) | 1.00 (ref.) | 1.00 (ref.) |
| *1 medication (N=18,790)* | 1,530 (8.1) | 1.01 (0.95-1.07) | 1.05 (0.99-1.11) |
| *2 medications (N=15,486)* | 1,299 (8.4) | 1.04 (0.98-1.10) | 1.07 (1.01-1.14) |
| *≥ 3 medications (N=40,456)* | 4,513 (11.2) | 1.37 (1.31-1.43) | 1.39 (1.33-1.45) |
| | | | |
| **Spontaneous preterm birth < 37 weeks** | | | |
| *0 medications (N=48,482)* | 2,212 (4.6) | 1.00 (ref.) | 1.00 (ref.) |
| *1 medication (N=18,790)* | 882 (4.7) | 1.03 (0.95-1.11) | 1.03 (0.95-1.11) |
| *2 medications (N=15,486)* | 790 (5.1) | 1.12 (1.03-1.21) | 1.11 (1.03-1.20) |
| *≥ 3 medications (N=40,456)* | 2,626 (6.5) | 1.39 (1.32-1.47) | 1.37 (1.29-1.45) |
| | | | |
| **Provider-initiated preterm birth < 37 weeks** | | | |
| *0 medications (N=48,482)* | 2,090 (4.3) | 1.00 (ref.) | 1.00 (ref.) |
| *1 medication (N=18,790)* | 767 (4.1) | 0.96 (0.89-1.04) | 1.00 (0.92-1.08) |
| *2 medications (N=15,486)* | 718 (4.6) | 1.08 (1.00-1.18) | 1.11 (1.02-1.21) |
| *≥ 3 medications (N=40,456)* | 2,742 (6.8) | 1.55 (1.46-1.64) | 1.51 (1.43-1.60) |
| | | | |
| **Any congenital anomaly** | | | |
| *0 medications (N=48,482)* | 2,809 (5.8) | 1.00 (ref.) | 1.00 (ref.) |
| *1 medication (N=18,790)* | 1,087 (5.8) | 1.00 (0.93-1.07) | 1.02 (0.96-1.10) |
| *2 medications (N=15,486)* | 895 (5.8) | 1.00 (0.93-1.07) | 1.02 (0.95-1.10) |
| *≥ 3 medications (N=40,456)* | 2,794 (6.9) | 1.19 (1.13-1.25) | 1.20 (1.14-1.26) |

Abbreviations: CI = confidence interval.

[a] Adjusted for maternal age, parity, immigrant/refugee status, neighbourhood income quintile, and rural residence.

© 2026 Brown HK et al. *JAMA Network Open*.

**eTable 6. Odds of 1 and ≥ 2 indicators of severe neonatal morbidity or mortality (SNM-M) in mothers with 0, 1, 2 or ≥ 3 pre-pregnancy chronic conditions.**

| **Study outcome *by number of chronic conditions*** | **Outcome of 1 SNM-M indicator[a]** | | **Outcome of ≥ 2 SNM-M indicators[a]** | |
|---|---|---|---|---|
| | **No. (%)** | **Adjusted odds ratio (95% CI)[b,c]** | **No. (%)** | **Adjusted odds ratio (95% CI)[b,c]** |
| *0 chronic conditions (N=458,019)* | 16,102 (3.5) | 1.00 (ref.) | 13,386 (2.9) | 1.00 (ref.) |
| *1 chronic condition (N=196,212)* | 8,794 (4.5) | 1.29 (1.25-1.32) | 7,120 (3.6) | 1.26 (1.22-1.29) |
| *2 chronic conditions (N=53,283)* | 2,987 (5.6) | 1.64 (1.57-1.71) | 2,467 (4.6) | 1.63 (1.56-1.70) |
| *≥ 3 chronic conditions (N=15,429)* | 1,084 (7.0) | 2.12 (1.98-2.26) | 948 (6.1) | 2.22 (2.08-2.38) |

Abbreviations: CI = confidence interval.

[a] Newborns with 0 SNM-M indicators form the outcome referent.
[b] Using multinomial logistic regression. One newborn per individual was randomly selected for this analysis to avoid any clustering effect.
[c] Adjusted for maternal age, parity, immigrant/refugee status, neighbourhood income quintile, and rural residence.

© 2026 Brown HK et al. *JAMA Network Open*.
