# Supplementary material for: Multiple Maternal Chronic Conditions and Risk of Severe Neonatal Morbidity and Mortality
Source: JAMA Netw Open. 2026 Jan 23;9(1):e2555558. doi: 10.1001/jamanetworkopen.2025.55558 (PMC12831157; doi:10.1001/jamanetworkopen.2025.55558)
Supplement: Supplement 2. — Data Sharing Statement [file jamanetwopen-e2555558-s002.pdf]

Data Sharing Statement

Brown. Multiple Maternal Chronic Conditions and Risk of Severe Neonatal Morbidity and Mortality. *JAMA Netw Open*. Published online January 23 2026. doi:10.1001/jamanetworkopen.2025.55558

Data
Data available: No

Additional Information
Explanation for why data not available: The dataset from this study is held securely in coded form at ICES. While legal data sharing agreements between ICES and data providers (e.g., healthcare organizations and government) prohibit ICES from making the dataset publicly available, access may be granted to those who meet prespecified criteria for confidential access, available at https://www.ices.on.ca/DAS/AHRQ (email: das@ices.on.ca). The full dataset creation plan and underlying analytical code are available from the authors on request, understanding that the computer programs might rely on coding templates or macros that are unique to ICES and are therefore either inaccessible or require modification.
